# Downregulation of Wnt signaling by sonic hedgehog activation promotes repopulation of human tumor cell lines

**DOI:** 10.1242/dmm.018887

**Published:** 2015-02-20

**Authors:** Jingjing Ma, Jin Cheng, Yanping Gong, Ling Tian, Qian Huang

**Affiliations:** 1 The Comprehensive Cancer Center & Shanghai Key Laboratory for Pancreatic Diseases, Shanghai First People’s Hospital, School of Medicine, Shanghai Jiao Tong University, Shanghai 201620, China.; 2 Department of Pharmacy, The First Affiliated Hospital of Soochow University, Suzhou 215006, China.; 3 Experimental Research Center, Shanghai First People’s Hospital, School of Medicine, Shanghai Jiao Tong University, Shanghai 201620, China.

**Keywords:** Colon cancer, Pancreatic cancer, Radiotherapy, Repopulation, Wnt signaling

## Abstract

Tumor repopulation after radiotherapy is a big obstacle for clinical cancer therapy. The molecular mechanisms of tumor cell repopulation after radiotherapy remain unclear. This study investigated the role of sonic hedgehog (SHH) and Wnt signaling pathways in tumor repopulation after radiotherapy in an *in vitro* repopulation model. In this model, irradiated dying tumor cells functioned as feeder cells, whereas luciferase-labeled living tumor cells acted as reporter cells. Proliferation of reporter cells was measured by bioluminescence imaging. Results showed that irradiated dying HT29 and Panc1 tumor cells significantly stimulated the repopulation of living cells in their respective cultures. In HT29 and Panc1 cells, radiation significantly inhibited Wnt activity. In the irradiated dying HT29 and Panc1 cells, the level of the activated nuclear β-catenin was significantly decreased. Treatment with the Wnt agonist 68166 significantly decreased, whereas treatment with Wnt antagonist significantly increased, repopulation in HT29 and Panc1 tumor cells in a dose-dependent manner. β-catenin short-hairpin RNA (shRNA) also significantly promoted tumor cell repopulation. The level of secreted frizzled related protein-1 (SFRP1), hedgehog and Gli1 were increased in irradiated cells. Our results highlight the interaction between Wnt and SHH signaling pathways in dying tumor cells and suggest that downregulation of Wnt signaling after SHH activation is negatively associated with tumor repopulation.

## INTRODUCTION

Tumor repopulation after radiotherapy is a challenge for clinical cancer therapy. During tumor repopulation, a few surviving tumor cells that escaped radiotherapy can regrow and repopulate at previously cleared regions. Tumor repopulation is a major cause for tumor relapse, and the rate of repopulation might increase over time (Withers et al., 1988). Although the activation of proliferation-related signaling pathways might be involved in tumor cell repopulation (Kim and Tannock, 2005), the associated molecular mechanisms have not been fully elucidated.

Wnt signaling plays important roles in carcinogenesis. Wnt signaling is initiated when Wnt ligands engage their cognate receptor complex. The central player of Wnt signaling is the cytoplasmic protein β-catenin, which accumulates in the cell cytoplasm, translocates into the nucleus and functions as a cofactor for transcription factors of the T-cell factor/lymphoid enhancing factor (TCF/LEF) family (Giles et al., 2003). When Wnt receptors are not engaged, β-catenin is destabilized (Reya and Clevers, 2005). Newly reported, non-canonical Wnts, such as Wnt4 and Wnt5a, can also induce β-catenin-dependent signaling (Ring et al., 2014). Interestingly, it has been reported that sonic hedgehog (SHH) signaling and Wnt signaling interact in tumors. For example, in colorectal cancer, overexpression of Gli1 (a downstream component of the SHH signaling pathway) inhibits Wnt signaling and colorectal cancer cell proliferation, even in cells possessing the stabilizing mutation of β-catenin (Akiyoshi et al., 2006). In neural progenitors, Wnt signaling and SHH signaling coordinately regulate cell cycle progression, with SHH signaling activation required upstream (Alvarez-Medina et al., 2009). Our recent study has demonstrated that SHH signaling activation is involved in tumor repopulation after radiotherapy (Ma et al., 2013), and Sims-Mourtada et al. has reported that SHH signaling pathway is extensively activated in residual tumors after chemo-radiotherapy and that blocking SHH signaling in esophageal cancer could enhance radiation cytotoxicity (Sims-Mourtada et al., 2006). However, Wnt signaling and its interaction with SHH signaling in dying tumor cells, as well as their roles in stimulating tumor cell repopulation, has not been reported.

In this study, we investigated the role of Wnt signaling in tumor repopulation. To do this, living tumor cells labeled by the firefly luciferase and green fluorescent protein fusion genes were seeded onto irradiated tumor cells to mimic tumor repopulation *in vitro*. Next, β-catenin level was measured in nuclei, SHH and Gli1 levels were measured in irradiated tumor cells, and the effects of Wnt agonist, Wnt antagonist and β-catenin shRNA on tumor repopulation were investigated.

## RESULTS

### Luciferase activity in reporter cells is linearly associated with cell numbers

**Fig. 1.**
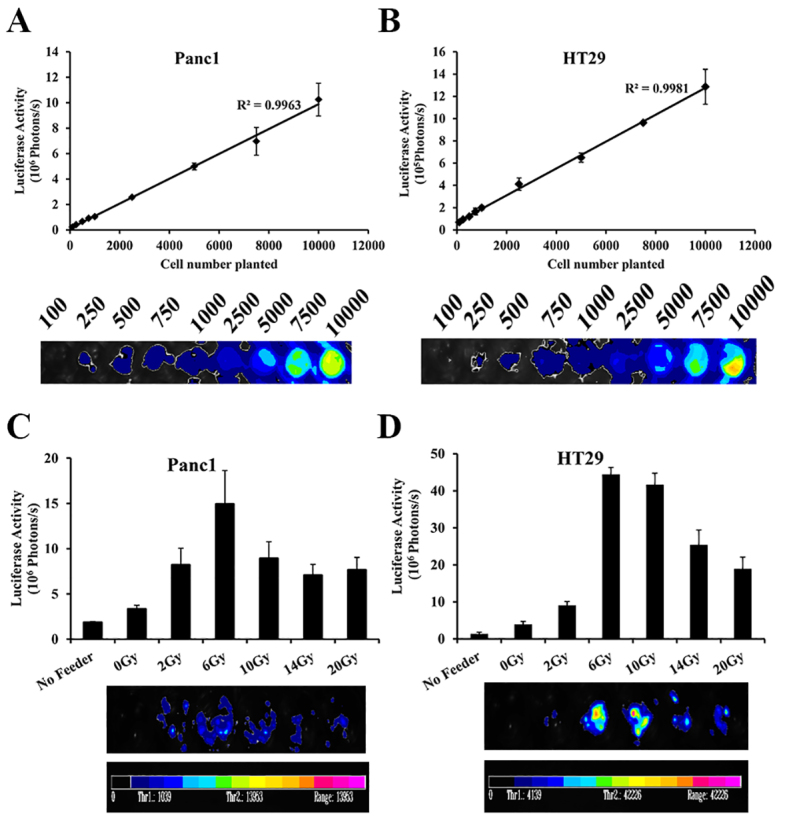
**Growth-stimulating effects of irradiated tumor cells on live tumor cell growth.** (A) Correlation analysis of photons measured by bioluminescence imaging with the number of Fluc-labeled Panc1 cells. The average number of photons from three repeats for each cell number were analyzed. Lower panel: representative luciferase image of the different number of plated Panc1Fluc cells. (B) Correlation analysis of photons measured by bioluminescence imaging with the number of Fluc-labeled HT29 cells. Lower panel: representative luciferase images. (C) Analysis of signal intensity of Panc1Fluc cells grown on irradiated Panc1 cells. 2.5×10^5^ irradiated Panc1 cells were plated into 24-well plates as feeder cells. 1000 Panc1Fluc cells were plated into each well as reporter with or without feeder cells. After 14 days, the plate was imaged for bioluminescence intensity. Upper panel: luciferase activity analysis. Lower panel: representative bioluminescence images. (D) Analysis of signal intensity of HT29Fluc cells grown on irradiated HT29 cells. Upper panel: luciferase activity analysis. Lower panel: representative bioluminescence image. Results are mean±s.d.

Panc1 and HT29 tumor cells were labeled with a luciferase–EGFP fusion gene (denoted Panc1Fluc and HT29Fluc, respectively). The stably transduced Panc1Fluc and HT29Fluc cells were seeded in a 96-well plate, and luciferase activity was measured 24 hours later. As shown in Fig. 1A,B, the intensity of the bioluminescence signal (photons per seccond) was linearly correlated with the number of cells seeded (R^2^=0.9963 for Panc1Fluc cells; R^2^=0.9981 for HT29Fluc cells), suggesting that the intensity of bioluminescence signal can be used to represent the growth and relative numbers of reporter cells.

### Stimulation of living tumor cell growth by irradiated dying tumor cells

To mimic scenarios *in vivo*, ~1000 living Panc1Fluc cells or HT29Fluc cells (reporter cells) were seeded onto a bed of 2.5×10^5^ unlabeled homologous tumor cells (feeder cells) that had been irradiated at 2 Gy, 6 Gy, 10 Gy, 14 Gy or 20 Gy, respectively. The growth-stimulating effect of feeder cells on the living reporter cells was measured by bioluminescence imaging. As shown in Fig. 1C,D, the feeder cells irradiated with 6 Gy showed the highest stimulatory effect in both HT29 and Panc1 cells. For HT29Fluc cells, the signal in reporter cells grown on 6 Gy irradiated feeder was 32 times higher than reporter cells grown alone and 11 times higher than reporter cells grown on non-irradiated feeder cells. For Panc1 cells, the signal in reporter cells grown on 6 Gy irradiated feeder was eight times higher than reporter alone and four times higher than reporter cells grown on non-irradiated feeder cells. Furthermore, cells irradiated with higher doses than 6 Gy showed less stimulatory effect on living cell growth in both HT29 and Panc1 cells.

**TRANSLATIONAL IMPACT**

**Clinical issue**

Radiotherapy is widely used as an effective treatment for cancer. More than half of all individuals with cancer receive radiotherapy during the course of treatment. Tumor cell repopulation is one of the main causes of radiotherapy failure. However, its mechanism remains unclear. Research has shown that radioresistant cancer cells that survive radiation treatment induce tumor cell repopulation and recurrence. This study focused on the interaction that irradiated cells have with non-radiated cells, which is considered one of the causes of tumor repopulation after radiation treatment. This is an interesting topic of research, relevant for advancing our understanding of the full effects of radiotherapy.

**Results**

In this study, the effects of irradiated cells on non-irradiated cell populations were studied by a simple and clear *in vitro* model. In this model, irradiated cells worked as feeder cells, whereas non-irradiated living cells were labeled with luciferase to act as reporter cells. The irradiated cells and living cells were co-cultured. The population activity of living cells was measured by a bioluminescence image assay. Results showed that irradiated cells can promote non-irradiated living cell repopulation. Interestingly, the Wnt signaling pathway was downregulated and SHH (sonic hedgehog) signaling pathway was activated in irradiated feeder cells. Further results suggested that Wnt pathway downregulation in irradiated cells might be a result of increased secreted frizzled-related protein 1 (SFRP1) expression, which could be induced by SHH activation.

**Implications and future directions**

Theoretically, radiation is supposed to kill cancer cells by causing DNA damage, which leads to cell death. Hence, radiotherapy is commonly considered as a local cytotoxic treatment. However, tumors are nonhomogeneous cell masses, and different parts of the tumor might receive varying doses of radiation depending on their location in the radiation field. Few studies have focused on what happens between different cells receiving different doses of radiation. Data from the current study revealed that irradiated cells can promote growth and repopulation of non-irradiated cells. More interestingly, two major signaling pathways (Wnt and SHH) are simultaneously active in irradiated cells. These observations suggest that effects of radiation on cancer cells are very complicated and that, although inducing cell death, radiation might also indirectly be responsible for the regeneration of tumor populations. Although this *in vitro* model is a simple representation of the complex mechanism of tumor repopulation happening *in vivo*, the design is clear and appropriate for explaining the causes of tumor repopulation. Further translational studies using a similar *in vivo* model are necessary to confirm the current findings, which might help improve the efficacy of radiotherapy in cancer treatment.

### Wnt signaling pathway was downregulated in irradiated tumor cells

**Fig. 2.**
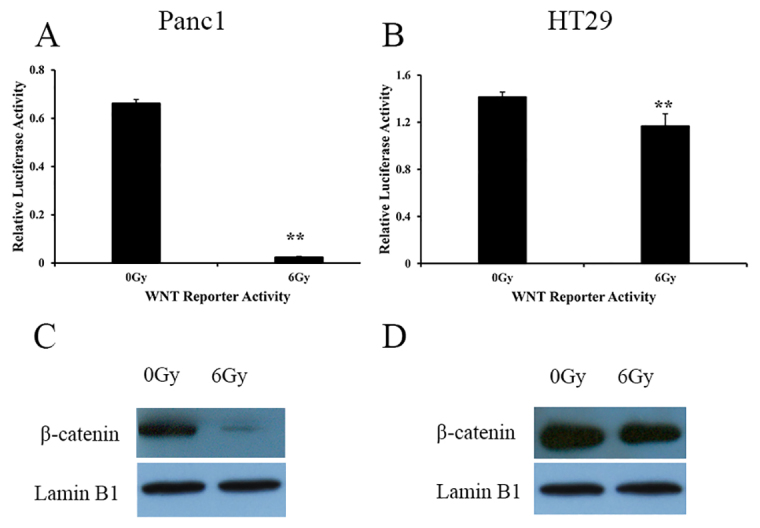
**Evidence for Wnt signaling pathway inhibition in irradiated Panc1 and HT29 cells.** (A) Wnt reporter luciferase activity in irradiated and non-irradiated Panc1 cells. **P<0.01. (B) Wnt reporter luciferase activity in irradiated and non-irradiated HT29 cells. **P<0.01. Results are mean±s.d. (C) Nuclear β-catenin protein levels in Panc1 cells that were irradiated at 6 Gy or were not irradiated. (D) Nuclear β-catenin protein levels in HT29 cells that were irradiated at 6 Gy or were not irradiated. Lamin B1 serves as a control.

To test whether the Wnt pathway was activated in irradiated tumor cells and its role in tumor repopulation, a 8×TopFlash luciferase reporter containing the wild-type LEF/TCF-binding site and a 8×FopFlash luciferase reporter containing a mutated LEF/TCF-binding site were stably transduced in Panc1 and HT29 cells. The luciferase activity was measured before and after 6 Gy irradiation. The relative luciferase activity was calculated by dividing the activity of the 8×TopFlash luciferase reporter with the activity of the 8×FopFlash luciferase reporter. Interestingly, the relative luciferase activity was significantly lower in irradiated tumor cells than in untreated tumor cells (*P*<0.01), suggesting that the activity of the Wnt pathway was decreased in cancer cells irradiated at 6 Gy. Although reduced Wnt activity was observed in both the irradiated Panc1 (Fig. 2A) and HT29 (Fig. 2B) cells, a more obvious decrease in Wnt activity was observed in Panc1 cells.

β-catenin is a major component of the Wnt pathway, and the translocation of β-catenin from the cytoplasm to the nucleus represents activation of β-catenin as well as the Wnt signaling pathway. The β-catenin level in the nucleus was assessed by western blotting. As shown in Fig. 2C,D, the amount of β-catenin in both irradiated Panc1 and HT29 cells was lower than in untreated control cells. The amount of β-catenin in irradiated Panc1 cells was much lower than in irradiated HT29 cells.

### Wnt activity affects growth of reporters

**Fig. 3.**
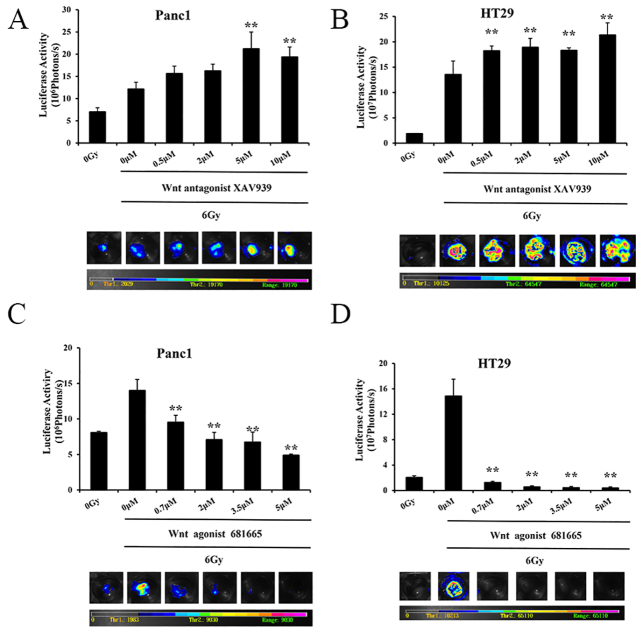
**Effects of Wnt signaling regulators on tumor cell repopulation induced by dying cells.** (A) XAV939 enhanced the Panc1Fluc cell growth induced by dying cells. Upper panel: signal intensity analysis from bioluminescence imaging. Lower panel: representative bioluminescence images. **P<0.01. (B) XAV939 stimulated HT29Fluc cell growth induced by dying cell. Upper panel: signal intensity analysis from bioluminescence imaging. Lower panel: representative bioluminescence images. **P<0.01. (C) Wnt agonist 681665 inhibits Panc1Fluc cell growth induced by dying Panc1 cells. Upper panel: signal intensity analysis from bioluminescence imaging. Lower panel: representative bioluminescence images. **P<0.01. (D) Wnt agonist 681665 inhibits HT29Fluc cell growth induced by dying HT29 cells. Upper panel: signal intensity analysis from bioluminescence imaging. Lower panel: representative bioluminescence images. **P<0.01. Results were obtained as described in Fig. 1 and are mean±s.d.

Panc1 cells were irradiated at 6 Gy and seeded as feeders in 24-well plates and cultured with medium containing or not containing XAV939 (a Wnt signaling inhibitor). The reporter cells grew more rapidly after treatment with 5 μM and 10 μM of XAV939 than did reporter cells without XAV939 (Fig. 3A, *P*<0.01). In HT29 cells, all tested XAV939 concentrations (0.5 μM to 10 μM) showed a stimulatory effect on the growth of reporter cells (Fig. 3B, *P*<0.01).

To further confirm the role of Wnt signaling pathway in irradiated tumor cells stimulating living tumor cell growth, the effect of the Wnt signaling agonist 68166 at 0.7 μM, 2 μM, 3.5 μM and 5 μM on reporter cell growth was tested. Panc1 cells were irradiated at 6 Gy and seeded as feeders. Wnt agonist treatment significantly decreased living Panc1Fluc tumor cell growth (*P*<0.01) in a dose-dependent manner (Fig. 3C). Similar results were observed in HT29Fluc cells (Fig. 3D). Wnt agonist treatment decreased living HT29Fluc cell growth more strongly than Panc1Fluc cells (*P*<0.01). Collectively, these results show that manipulation of Wnt activity can influence the growth of reporter cells stimulated by irradiated dying tumor cells.

### Knockdown of β-catenin enhances the stimulatory effect of dying cells

**Fig. 4.**
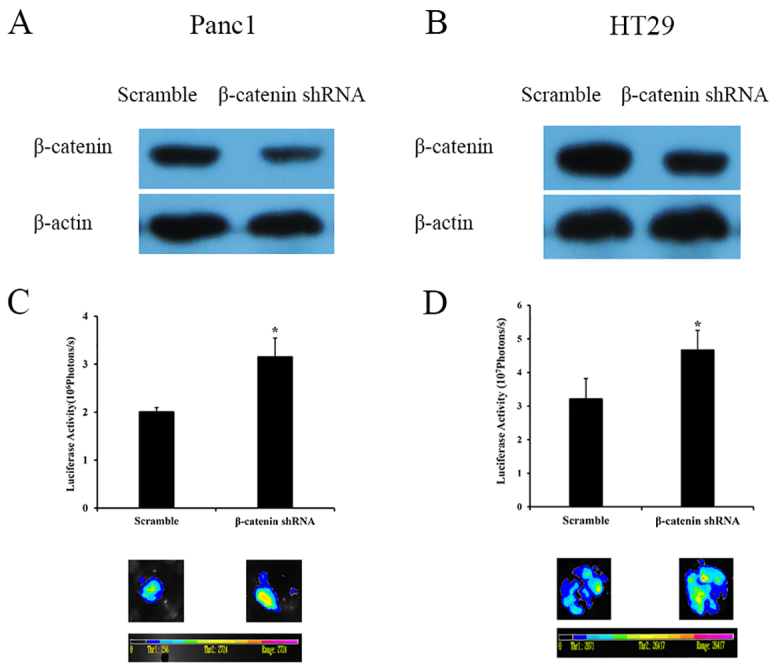
**Effects of β-catenin knockdown on tumor cell growth induced by dying cells.** (A) Western blot of β-catenin protein expression in β-catenin-shRNA-transduced Panc1 cells. (B) Western blot of β-catenin protein expression in β-catenin-shRNA-transduced HT29 cells. (C) The increased Panc1Fluc cell growth on dying β-catenin-shRNA-transduced Panc1 cells. Upper panel: signal intensity analysis from bioluminescence imaging. Lower panel: representative bioluminescence images. *P<0.05. (D) The increased HT29Fluc cell growth on dying β-catenin-shRNA-transduced HT29 cells. Upper panel: signal intensity analysis from bioluminescence imaging. Lower panel: representative bioluminescence images. *P<0.05. Results were obtained as described in Fig. 1 and are mean±s.d.

Knockdown of β-catenin expression was performed in Panc1 and HT29 cells (Fig. 4A,B) by stable transduction of β-catenin short hairpin RNA (shRNA). Panc1 and HT29 cells with or without β-catenin knockdown were irradiated at 6 Gy as feeder cells. The growth of living Panc1Fluc or HT29Fluc cells seeded onto β-catenin knockdown feeder cells was significantly enhanced compared with controls transduced with scrambled shRNA (*P*<0.05) (Fig. 4C,D).

### Inhibition of Wnt signaling in irradiated dying cells is associated with the activation of SHH signaling

**Fig. 5.**
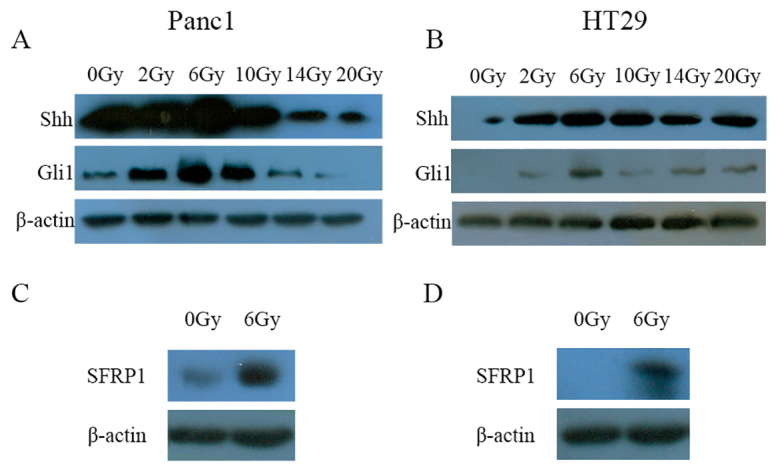
**SHH signaling activation in irradiated cells.** (A) Representative western blot of SHH and Gli1 proteins in Panc1 cells irradiated at the indicated dose. (B) Representative western blot of SHH and Gli1 proteins in HT29 cells irradiated at the indicated dose. (C) Western blot showing increased SFRP1 in irradiated Panc1 cells after radiation with 6 Gy in comparison with non-irradiated cells. (D) Western blot showing increased SFRP1 in irradiated HT29 cells after radiation with 6 Gy in comparison with non-irradiated cells.

A previous study has demonstrated that SHH signaling is activated in irradiated dying tumor cells, and that the activation of SHH in irradiated dying cells is positively associated with the stimulatory effect on living tumor cell growth (Ma et al., 2013). As shown in Fig. 5A,B, the protein level of SHH (ligand) and Gli1 (a transcription factor that can activate the transcription of SHH target genes) were increased in irradiated tumor cells with a peak at 6 Gy.

Secreted frizzled related protein-1 (SFRP1) is the most likely SHH target that confines canonical Wnt signaling (Katoh and Katoh, 2006). The expression level of SFRP1 in Panc1 cells and HT29 cells that were not irradiated or were irradiated with 6 Gy was assessed by western blotting. Increased SFRP1 expression was observed in Panc1 cells and HT29 cells irradiated with 6 Gy (Fig. 5C,D).

## DISCUSSION

In the clinic, a few surviving tumor cells that escape from exposure to radiotherapy or chemotherapy can rapidly repopulate (Kessenbrock et al., 2013; Thompson et al., 2011). This process is nearly a universal obstacle in cancer treatment and a major contributor to treatment failure. However, its underlying mechanism is poorly understood. In this study, we demonstrated that activated SHH and inactivated Wnt signaling in irradiated tumor cells play a crucial role in the repopulation of colon cancer and pancreatic cancer cells.

Cancer stem cells are classically resistant to conventional chemotherapy and radiation therapy through a variety of mechanisms, including the effects of tumor cell repopulation (Moncharmont et al., 2012). According to Katoh’s summary, Wnt, FGF, Notch, SHH and TGFβ/BMP signaling cascades constitute the stem-cell signaling network, playing a key role in the maintenance or homeostasis of cancer stem cells (Katoh, 2011). The Wnt signaling pathway plays an important role in regenerative proliferation and stem cell maintenance (Katoh and Katoh, 2007; Kessenbrock et al., 2013). However, the role of Wnt signaling in the effect of dying tumor cells on tumor cell repopulation has not been reported. In this study, the role of Wnt pathway in tumor repopulation and its relationship with SHH signaling pathway was investigated in colon cancer and pancreatic cancer cells. Interestingly, the expression of β-catenin, an important component of Wnt signaling pathway, was remarkably decreased in irradiated tumor cells. Moreover, the stimulatory effect of dying cells could be further inhibited by Wnt signaling agonists, but this effect was enhanced by Wnt signaling antagonists or knockdown of β-catenin. However, a previous study has suggested that β-catenin-positive hepatocytes exhibit growth and survival advantages that allow them to repopulate β-catenin-null livers (Thompson et al., 2011). Furthermore, expression of active β-catenin enhances self-renewal of Sca1+ cells, which have been identified as the primary mammary gland progenitor cells, whereas suppressing β-catenin decreases self-renewal of the Sca1+ cells (Chen et al., 2007). Therefore, the Wnt pathway has been recognized as an attractive target for directed anti-stem-cell and anti-self-renewal therapeutics (Behbod and Rosen, 2005). We hypothesized that Wnt signaling is activated after radiation and that it negatively associated with tumor cell repopulation. However, our finding is in contrast to the usual stimulating role of Wnt signaling during tumorigenesis, because in our model Wnt signaling was inactivated, and inactivated Wnt signaling stimulated tumor cell repopulation. This might suggest that the role of Wnt signaling pathway is complicated and that different Wnt ligands function in different ways. It is worthwhile mentioning that the recently identified Wnt5a might increase hematopoietic stem cell repopulation by antagonizing the canonical Wnt pathway (Nemeth et al., 2007).

Our previous study demonstrated that SHH was activated in dying cells and that the manipulation of SHH signaling could influence tumor repopulation (Ma et al., 2013). However, the interaction between SHH signaling and Wnt signaling in dying tumor cells and its role in tumor cell repopulation had not been elucidated. During bladder injury, SHH expression in the basal cells within the urothelium increases and elicits increased stromal expression of Wnt protein signals, which in turn stimulate the proliferation of both urothelial and stromal cells. In this case, SHH and Wnt positively interact to help injury-induced epithelial regeneration, and this suggests that there is a conceptual framework for injury repair in endodermal organs (Shin et al., 2011). In intestinal epithelium, SHH represses canonical Wnt signaling, which restricts the expression of genes targeted by Wnt. SFRP1 acts as a secretory Wnt antagonist (Reins et al., 2010) and might be the link between Wnt and SHH signaling pathways. SFRP1 is also able to downregulate Wnt signaling by forming an inhibitory complex with the Frizzled receptors. Furthermore, SHH secreted from differentiated epithelial cells can induce SFRP1 expression in mesenchymal cells, which keep differentiated epithelial cells away from the effects of canonical Wnt signaling. This indicates that SFRP1 is a SHH target that helps confine canonical Wnt signaling to within stem or progenitor cells (Katoh and Katoh, 2006). In addition, in gastric cancer cells, the ectopic expression of Gli1 decreases nuclear β-catenin staining (Kim et al., 2010). The Gli-binding site was identified within the human SFRP1 (Kim et al., 2010). We therefore deduced that SFRP1 might be the link between Wnt and SHH signaling pathways during tumor repopulation. The present study revealed a significant increase in SFRP1, SHH and Gli1 expression in irradiated tumor cells. This could explain why the Wnt signaling pathway is inhibited in these cells. The activated SHH might be responsible for the dying cells stimulating living tumor cell growth, and SHH signaling might act upstream of the Wnt signaling pathway. Finally, whether the interaction between SHH signaling and Wnt signaling causes an enhancing or suppressing effect might depend on various conditions.

**Fig. 6.**
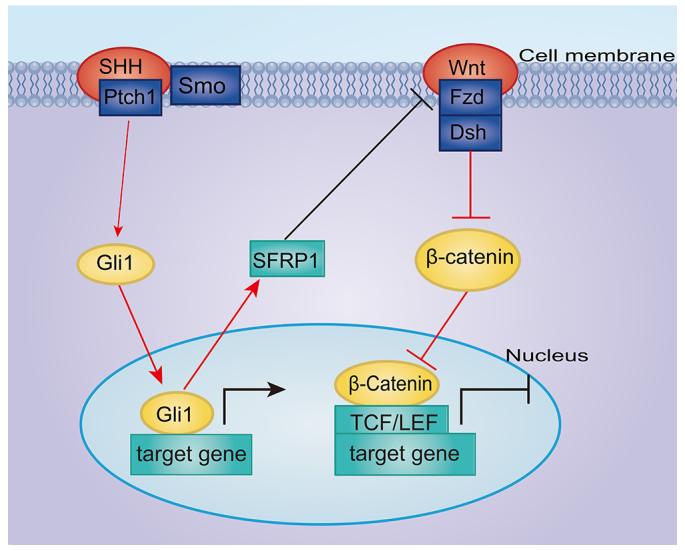
**A schematic of the effects of SHH signaling activation and Wnt signaling inhibition on living tumor cell growth stimulated by radiated dying tumor cells *in vitro*.** After radiation, SHH signaling is activated, which in turn upregulates the expression of the signaling molecule SFRP1. SFRP1, as a secreted-type Wnt antagonist, downregulates Wnt signaling. Consequently, living tumor cells are stimulated and acquire accelerated repopulation. Red lines represent the finds in this study and black lines represent information from the published literature.

In summary, our results provide a new understanding of the complicated crosstalk between the SHH and Wnt signaling pathways during tumor cell repopulation (Fig. 6), which might play an important role in tumor growth and relapse after radiotherapy or chemotherapy. Given that tumor repopulation is a major cause of treatment failure, one of the practical implications of this study is that Wnt signaling inhibitors should be carefully used in cancer therapy, especially in radiotherapy, because Wnt signaling inhibitors might enhance tumor cell repopulation. Our results also point to the potential efficacy of adjuvant use of SHH signaling inhibitors during radiotherapy.

## MATERIALS AND METHODS

### Cell culture

Human pancreatic cancer cell line Panc1 and human colon cancer cell line HT29 were purchased from the Chinese Academy of Science (Shanghai, China) and cultured in Dulbecco’s modified Eagle’s medium (DMEM) (Thermo Scientific Inc., Beijing, China) with 10% fetal bovine serum (Tianhang Biological Technology Co., Ltd, Hangzhou, China), 100 U/ml penicillin, and 100 μg/ml streptomycin at 37°C in a humidified atmosphere containing 5% CO_2_.

### Gene transduction and selection of stably transduced cells

The exogenous gene was introduced into the target cells using a lentiviral vector derived from the pLEX system (Thermo Scientific Inc.), containing a puromycin-resistance gene. The following genes were cloned into the lentiviral vector: a firefly luciferase and green fluorescent protein fusion gene (Fluc), driven by the CMV promoter; a Wnt reporter gene and its control [i.e. 8×wild-type LEF/TCF-binding sites (8×TopFlash luciferase reporter that can bind β-catenin) and 8×mutated LEF/TCF-binding sites (8×FopFlash luciferase reporter, which cannot bind β-catenin)]; and shRNA against β-catenin or scrambled shRNA. All lentiviral vectors were packaged in 293T cells by following the manufacturer’s instructions. HT29 or Panc1 cells were infected by the lentivirus and then the stably transduced cells were selected with 2 μg/ml puromycin for 2 weeks.

### shRNA and knockdown of gene expression

HT29 or Panc1 cells were infected using lentivirus containing shRNA against the β-catenin-encoding gene (5′-TGCTTGGTTCACCAGTGGATT-3′) or scrambled sequence (5′-GCCTAAGGTTAAGTCGCCCTCG-3′). The stably transduced cells were selected with 2 μg/ml puromycin for 2 weeks. Silencing efficiency was confirmed by western blotting.

### Tumor repopulation model *in vitro*

HT29 cells or Panc1 cells cultured in 10-cm Petri dishes were irradiated with X-rays using an Oncor linear accelerator (Siemens, Amberg, Germany). The dose rate of the machine is about 3.6 Gy per minute and the dose was given as indicated. The dose that showed the most stimulatory effect was chosen for further study. At 24 hours after irradiation, tumor cells were trypsinized and then seeded into 24-well plates at a density of 2.5×10^5^ cells per well in triplicate. After another 24 hours, 1000 Fluc-labeled living tumor cells were seeded per well as a reporter. DMEM with 2% FBS was used, and the medium was changed every 2 days for 14 days. The growth of reporter cells was evaluated by a bioluminescence imaging method.

### Wnt reporter and luciferase activity

The luciferase reporter assay system (E1500, Promega, WI) was used to measure firefly luciferase activity according to the manufacturer’s instructions. Panc1 and HT29 cells expressing the 8×TopFlash luciferase reporter or 8×FopFlash luciferase reporter were irradiated with 6 Gy of ionizing radiation. The non-irradiated cells were used as a control. The luciferase activity in cells irradiated with 6 Gy was measured with a Berthold luminometer (Berthold Technologies, Bad Wildbad, Germany). The luciferase activity in the treatment group was normalized to the value in cells expressing the FopFlash luciferase reporter. All tests were performed in triplicate and repeated three times.

### Bioluminescence imaging

Bioluminescence images were taken with the NC100 instrument from Berthold technologies (Bad Wildbad, Germany). Briefly, the culture medium was removed and then D-luciferin (0.15 mg/ml in PBS, Promega, WI) was added to each well. After 5 minutes, images of the cells were taken to detect the luciferase signals, and the signal intensity was quantitatively analyzed by the manufacturer’s supplied software. Images were taken at around the same experimental day to minimize variability. For the Panc1 or HT29 *in vitro* repopulation model, images were usually taken at day 14.

### Antibodies and key chemicals used in this study

Primary antibodies against β-catenin, sonic hedgehog (SHH), glioma-associated oncogene 1 (Gli1) and β-actin were from Cell Signaling Technology (Boston, MA); antibody against secreted frizzled-related protein 1 (SFRP1) was from Epitomics; and secondary antibody conjugated to horseradish peroxidase (HRP) was from Bio-Rad. Wnt signaling antagonist XAV939 was obtained from Tocris Bioscience (Bristol, UK) and Wnt agonist 681665 was purchased from Merck Millipore (Darmstadt, Germany).

### Wnt agonist and antagonist treatments

Wnt signaling antagonist XAV939 is an inhibitor of tankyrase 1 and tankyrase 2, which can stimulate β-catenin degradation by stabilizing axin (Tung et al., 2013). Wnt agonist 681665 is a cell-permeable pyrimidine compound that acts as a potent and selective activator of Wnt signaling without inhibiting the activity of GSK-3β. XAV939 and Wnt agonist 681665 were added immediately as feeder when irradiated Panc1 or HT29 cells were seeded into 24 well plates. The 0.5 μM, 2 μM, 5 μM and 10 μM Wnt antagonist XAV939 and 0.7 μM, 2 μM, 3.5 μM and 5 μM Wnt agonist 681665 were used. The medium was changed every 48 hours and replaced with fresh medium containing the corresponding concentration of XAV939 or Wnt agonist 681665. The growth of living HT29Fluc or Panc1Fluc reporter cells was monitored 14 days later by bioluminescence imaging.

### Western blot analysis

Cells were washed twice with ice-cold PBS and lysed using 120–200 μl standard RIPA buffer containing protein inhibitors (Beyotime, Jiangsu, China). Protein in loading buffer was denatured by heating at 100°C for 10 minutes and then separated on a SDS-polyacrylamide gel by electrophoresis. 40–60 μg of protein per lane was loaded. The proteins were transferred onto a PVDF membrane (Bio-Rad, CA). The membranes were incubated in primary antibodies overnight at 4°C and then incubated in HRP-conjugated secondary antibodies for 2 hours at room temperature. The signals were visualized with ECL Plus substrates (Roche, Basel, Switzerland).

### Statistical analysis

Data were analyzed using SPSS 13.0 (SPSS, Inc., Chicago, IL). One-way analysis of variance (ANOVA) was used to assess statistical significance. *P*<0.05 was considered statistically significant.
